# Everybody's Page

**Published:** 1891-03-28

**Authors:** 


### Everybody's Page.

George Selwyn took such delight in seeing executions that he never missed being present at any which took place at Tyburn. When Lord Holland was confined to bed by dangerous illness, he was informed by his servant that Mr. Selwyn had recently called to enquire for him. "On his next visit," said Lord Holland, "be sure you let him in, for if I am alive I shall have great pleasure in seeing him, and if I am a corpse he will have great pleasure in seeing me."

These hat-pin accidents to the fair sex which we have been hearing about lately, ought not to be so lightly disregarded, for, after all, what a deadly weapon a hat-pin is, six inches of steel so placed as to be ready to pierce the head at any moment! At the best, hat-pins are poor means for keeping the hat on the head; yet strings, though useful, are not ornamental with every kind of hat, and then the only substitute that remains would be hats that *fit* the head. This again would entail dressing the hair in a very simple manner, and fashion, that all-powerful ruler, puts her veto on any nonsense of that sort, so hat-pins hold their own.

Sir Thomas Watson, Bart., M.D., in the early part of his London practice, was fetched to a house near Portland Place, where a gentleman had shot himself. He found the patient seated in an armchair in a well-furnished room. The bullet had entered at the forehead, and merely passed round the skull under the skin and gone out. Sir Thomas did what was required, and afterwards asked the patient what urged him, who appeared to be in health and affluence, to try to end his life. He replied, "I am tired of eating and drinking, dressing and undressing." An original way of trying to kill time, but not one to be generally recommended.

Of the coolness of the Great Duke of Wellington under the most trying circumstances Colonel Gurwood gives the following instance: The Duke was once in great danger of being drowned at sea. It was bed-time when the captain of the vessel came to him and said, "It will soon be over with us." "Very well," answered the Duke, "then I shall not take off my boots."

Now that the punishment of the cane, except for very grave offences, has disappeared from our schools, ordering boys to write out lines during the play hours appears to have taken its place. Is this a wise substitute? The same work is being done as during the other part of the day, and instead of being in the playground developing his body, the boy is in the schoolroom, leaning over his desk, scribbling lines, injuring his health, and spoiling his handwriting. It would be far better to give extra drills, make him march up and down the playground while the other boys are at cricket or football. The punishment would be as great, and no hindrance would be caused to his physical development.

Brotherly love will and should stand many tests, but it is doubtful if many men will submit to the self-sacrifice asked of them by the medical staff of a lodge of Knight Templars in America. According to an American paper, one hundred Knight Templars were asked to submit to the removal of a portion of their skin for the purpose of covering a large wound, the result of an operation for cancer, on one of their brother Templars, healthy skin from other bodies being considered necessary to bring about the desired healing. It is sad to find that the requisite number of Knights did not respond with the desired alacrity to such an appeal, which would have caused so little discomfort to themselves and such an undoubted boon to their afflicted brother!

